# Medicine

**Published:** 1907-03

**Authors:** J. R. Charles


### Progress of the Medical Sciences.

### MEDICINE.

**On the blood-glands as pathogenic factors in the production of diabetes.**—Lorand,[1] in a paper on this subject, points out that a certain antagonism exists between diabetes and tuberculosis in so far that tuberculous people rarely become diabetic or gouty, although diabetic patients may become tubercular. Conditions which are associated with thyroid deficiency seem to possess a certain immunity to gout and diabetes. Bayon, of Wurtzburg,[2] and de Querrain have carried out researches which show that in all grave infectious diseases the thyroid is in a condition termed by them "thyroiditis simplex." Roger and Garnier[3] had previously demonstrated the hypersecretion of the thyroid in infectious diseases, which hypersecretion in its turn might be followed by exhaustion. In many infectious diseases symptoms arise which indicate an increase in the function of the thyroid, such as hyperthermia, tachycardia, slight exophthalmos, perspiration, and occasionally diarrhœa and diuresis. Those who inherit a good working thyroid will have a greater immunity against infectious diseases than others. Children who become diabetic are, as a rule, of bright intelligence, a symptom attributed by the author to a condition of hyperthyroidea, in contrast to the mental torpor met with in conditions of athyroidea. The sexual glands are in close relationship with the other blood-glands, especially the thyroid, which frequently shows enlargement from overfunction, as in repeated pregnancy, prolonged lactation, &c. This overfunction may be followed by exhaustion leading to myxœdema. The islands of Langerhans, like the parathyroids and adrenals, also represent blood-glands. The ordinary secreting tissue of the pancreas may be destroyed by cirrhosis without any involvement of the islands, as in an example in the Vienna Pathological Institute, in which case there was no diabetes. The author, however, considers that the absence of any pathological change in the islands of Langerhans after death in cases of diabetes may be explained by defective functionating properties of the

[1] A. Lorand (Carlsbad), *Tr. Path. Soc. Lond.*, 1906, lvii. 1.
[2] *Wurzburger Abhandlungen*, 1904. [3] *Presse méd.*, 1899.

cells, the nervous tract to the pancreas possibly being involved; for, as he points out, Pawlow has demonstrated in the dog that there exists not only a psychic gastric juice, but also a psychic pancreatic juice. Sobolew found that in animals the islands diminish in size after the administration of rich carbohydrate food, a fact which may explain why diabetes may occur more readily in those who for a long time have been on carbohydrate diet. The various blood-glands stand in close relationship one with another, changes in one being followed by changes in others. In acromegaly diabetes is frequent. In this disease the author maintains that the thyroid is altered even more frequently than the hypophysis. The changes in the latter gland he regards as secondary to those in the former. He has shown that diabetes only occurs in those cases of acromegaly which show symptoms of hyperthyroidea. While on the one hand certain blood-glands are found degenerated in diabetes, on the other the extracts of other blood-glands may produce glycosuria or diabetes. Thus the injection of adrenal extract may produce considerable glycosuria. The extract of thyroid may produce even in a higher degree than adrenal extract considerable glycosuria, and even true diabetes. According to Naunyn, Van Noorden and Strauss, diabetes only follows in such cases where there is inherited predisposition. All the symptoms of the diabetes may, however, be produced by the administration of thyroid extract. In a patient suffering from acromegaly to whom the author administered thyroid tablets, glycosuria associated with the excretion of diacetic acid and acetone had existed for eight years. Before the administration of thyroid he had no glycosuria. There is an unusual tendency to alimentary glycosuria in ordinary cases of Graves's disease, although it is rare in those cases which are passing into a condition of myxœdema. In those cases in which diabetes has been noted in myxœdema it has apparently been produced by treatment with large doses of thyroid extract. The glycosuria of infectious diseases, and that after mental emotions, is probably related to the condition of hyperthyroidea set up in these states. Similarly, the increase of glycosuria during menstruation, and the lactosuria of lactation are also probably related to hyperthyroidea. Further, Hürthle has demonstrated that stagnation of bile causes an increase of thyroid colloid, and Gans, Finkler and Exuer have found glycosuria during biliary colic. It is an interesting fact that after thyroidectomy in the goat the milk becomes poor in sugar but rich in fats. The diminution of glycosuria before death in opium poisoning, its rarity in chronic tuberculosis and cancer the author attributes to a diminution of thyroid secretion in these conditions. There have been cases of diabetes in which the sugar has disappeared after the onset of tuberculosis. The auther has observed enlargement of the

[1] *Deutsche med. Wchnschr.*, 1897, No. 12.

thyroid in cases of diabetes, although he does not regard this as necessarily proving overaction of the gland. On examining the thyroid of three dogs in the laboratory of Prof. Minskowsky, of Cologne, after removal of the pancreas, he found much enlargement of the vesicles and much colloid material. The changes in the thyroid of these dogs very much resembled the changes produced in the thyroids of fowls after meat feeding by Chalmers Watson.[1] In the early stages of Graves's disease similar changes are found in the thyroid. In Graves's disease the increased secretion constitutes a toxic agent, causing, as shown by Magnus-Levy, a decomposition of albuminous substances. This decomposition leads to the splitting off of the carbohydrate radicle, the existence of which in the albuminous molecule has been demonstrated by Pavy.[2]

If under these circumstances the pancreas happens to be in any way degenerate, sugar appears in the urine. As an example of this, in the wards of Van Noorden was a case of Graves's disease in which after a few years diabetes developed. After death calculi were found in the pancreatic duct, and the gland tissue itself was degenerated. The author concludes that there are two important factors in diabetes: (1) degeneration of the pancreas; (2) hyperactivity of the thyroid. If the pancreas alone is degenerated the diabetes will be a light one, as in the diabetes of old people due to arterio-sclerosis of the pancreas, without any corresponding hyperactivity of the thyroid, for, as pointed out by Victor Horsley, the thyroid undergoes degenerative changes in old age. In young people the diabetes is more severe, because their thyroids are in good working order. The author considers that antithyroidin serum is capable of diminishing glycosuria, though he does not advocate its use in advanced cases. Diabetes will more readily occur in persons who take much meat, especially if they take large quantities of carbohydrates. Chalmers Watson has recently shown experimentally that an increased activity of the thyroid follows a meat diet.

**The acetone bodies.**[3]—Diacetic acid is so readily decomposed into acetone and carbonic acid, that no satisfactory method has been devised of separating the acetone and diacetic acid quantitatively in the urine. B-oxybutyric acid can, however, be separately estimated. While small amounts of acetone may be found in the urine of comparatively healthy individuals, the excretion of B-oxybutyric acid and diacetic acid are invariably due to pathological conditions. According to Schwartz, as much as 70% of the acetone which is excreted in pathological states may be excreted by the breath, while Geelmuyden has estimated that 80—95% may be lost through the lungs. There

[1] *Lancet*, 1905, i. 347 *et seq.* [2] Pavy, *Physiology of Carbohydrates*, 1894.

[3] "The Acetone Bodies: their Occurrence and Significance in Diabetes and other conditions," T. Stuart Hart, *Am. J. M. Sc.*, 1906, cxxxii.

is no constant ratio between the amount of acetone which is excreted by the kidneys and that excreted by the lungs. The acetone bodies are formed in considerable quantities in digestive disturbances and in febrile diseases. Certain drugs increase acetone production, including benzol, antipyrin, morphine and heroin. Nervous shock and excitement have been shown experimentally to lead to an increase of excretion of acetone bodies. An effort has been made, unsuccessfully however, to find out the particular organ of the body in which the acetone bodies are manufactured. In no organ, however, has acetone been found in larger amount than could be brought to it by the circulating blood. In the laboratory the acetone bodies have been derived from proteids, carbohydrates and fats. Almost certainly in the body an abnormal destruction of fat is the main source of acetone body formation. The amount of proteid destruction as represented by the output of nitrogen, phosphates and sulphates, and the amount of the acetone bodies excreted are not parallel either in diabetes or starvation. Magnus-Levy has reported a case in which there was a metabolism of 262 grammes of proteid in three days, and yet this patient excreted 342 grammes of B-oxybutyric acid in this period, which was more than could have been derived from the proteid. The withdrawal of carbohydrates from the diet of a normal man almost always results in an increase of acetone excretion, while the administration of carbohydrates has the opposite result. In some cases of diabetes the amount of B-oxybutyric acid varied directly with the amount of fat in the food. Large quantities of fat administered to healthy people in a mixed diet increase the excretion of acetone. An abnormal amount of acetonuria is not produced in starvation merely by diminishing the caloric value of the food. For example, a diet consisting of 1.4 litres of beer with 750 grammes of bread gave rise to no acetonuria. On the other hand, a pure proteid diet in a healthy man may cause considerable acetonuria, in which case the acetone is probably derived from the breaking down of the body fat. A pure fat diet may produce more acetonuria than starvation, the acetone in this case being derived both from the body and food fats. The amount of the acetone excretion depends to some extent on the character of the fat used. Addition of carbohydrate to any of the foregoing diets diminishes the acetone output. This reduction is exceedingly rapid, indicating that the first effect must be to furnish the conditions necessary for the complete oxidation of the acetone bodies at that time circulating in the blood. There is no parallelism between the excretion of sugar and the acetone bodies in diabetes. In this disease the main source of the acetone bodies is the imperfect metabolism of fat, which may be either the body or the food fat. As in health, the addition of carbohydrate to the diet generally results in a diminution of the acetone output, while its withdrawal may increase the acetone excretion. However, in advanced cases there may be exceptions to this statement. Fat feeding appears to be a frequent cause of the increase of acetone bodies. Fever in the diabetic usually reduces the output of sugar, while it increases that of the acetone bodies. The loss of power of oxidation in the diabetic has been shown by Waldvogel, who injected B-oxybutyric acid subcutaneously in cases of mild diabetes, and found that the resulting acetonuria was considerably greater than in his control experiments in healthy men. In coma, while B-oxybutyric acid is always present, acetone and diacetic acid may be relatively diminished, and sugar may be absent. The author considers that diabetes should be regarded as a disease in which there is a failure of the organism not only to utilise carbohydrates, but also to utilise fats. He believes that the disorders of fat metabolism are more than a secondary result of the diabetic's inability to assimilate carbohydrates, and that there is a direct perversion of the metabolism of fat. From the therapeutical aspect, since nervous irritation, excitement, hunger and fever all favour the production of the acetone bodies, they should as far as possible be avoided. Similarly, general anæsthetics should be avoided, and, if absolutely necessary, the starvation before and after should be a minimum. The withdrawal of carbohydrates should be carried out gradually, and the amount of fat which can be used without setting up acetonuria should be carefully watched. In selecting fatty foods, those containing the higher fatty acids should be chosen, since those containing the lower acids increase more markedly the production of the acetone bodies. If in spite of these precautions diacetic acid and B-oxybutyric acid are found, we must resort to the administration of alkalies. Simon, of Carlsbad,[1] has met with success in removing acetonuria in three cases by giving Parmesan cheese in amounts up to 3½ ounces daily. He recommends this cheese in cases where the exclusion of fats, particularly butter, has been unsuccessful in relieving this condition.

J. R. CHARLES.

### SURGERY.

[1] "Treatment of Acetonuria of Diabetes by the Ingestion of Parmesan Cheese," *La Semaine méd.*, 1905, No. 36.

